# The impact of bankruptcy regimes on entrepreneurship and innovation. Is there any relationship?

**DOI:** 10.1007/s11365-021-00773-3

**Published:** 2021-12-14

**Authors:** Błażej Prusak, Sylwia Morawska, Michał Łukowski, Przemysław Banasik

**Affiliations:** 1 Faculty of Management and Economics, Gdańsk University of Technology, Gdańsk, Poland; 2 SGH Warsaw School of Economics, Collegium of Business Administration, Warsaw, Poland; 3 Department of Investment and Financial Markets, Poznań University of Economics and Business, Poznań, Poland; 4 Faculty of Management and Economics, Gdańsk University of Technology, Gdańsk, Poland

**Keywords:** Entrepreneurship, Innovation, Bankruptcy, Law & Economics, Institutional economics, K22, L26, G15, G33

## Abstract

The literature review indicates that bankruptcy law may play an important role in and be one of the factors influencing the development of entrepreneurship, innovation, and thus economic growth, among other things. In previous studies, the analysis of the impact of bankruptcy law on individual variables has been conducted independently. Our aim was to conduct a holistic analysis, taking several factors into account simultaneously. Therefore, a descriptive model was proposed, based on which the following research hypothesis was formulated: *In countries characterised by an effective legal system and at the same time debtor-friendly bankruptcy law, the level of risk acceptance among entrepreneurs is higher, which is reflected in higher levels of entrepreneurship and innovation.* Based on the selected variables, a cross-sectional analysis was conducted using linear models estimated on the basis of the least-squares method. Additionally, to strengthen the conclusions drawn, the models were assessed in such a way enabling the analysis of causality as defined by Granger based on the two-step process. The results obtained allowed us to confirm the research hypothesis: in countries characterised by an efficient legal system and at the same time debtor-friendly bankruptcy law, the level of risk acceptance among entrepreneurs is higher, which is reflected in higher levels of entrepreneurship and innovation. The research results are particularly important from the point of view of legislators who are responsible for drafting amendments to bankruptcy law. Including certain debtor-friendly provisions may, in the long run, lead to increased entrepreneurship and innovation, and thus economic development.

## Introduction

Economic development depends on innovation and entrepreneurship (Hodgson, 2019). Innovation is crucial not only for national economies (Cieślik et al., 2016; Kraftova & Kraft, 2018; Sell, 2020; Tidd, 2006), but also for overcoming the contemporary challenges of fighting COVID-19 (Khan et al., 2021) and stagnation (Estrada et al., 2021). Significant emphasis is also placed on actions supporting innovation and entrepreneurship in the countries of the European Union. The need for such actions was recognised, among other things, in the Europe 2020 strategy and its "Innovation Union" initiative (European Commission, 2010). The renewed European research and innovation agenda included a number of actions to boost innovation in Europe and ensure sustainable prosperity (European Commission, 2018).

However, in order for innovation and entrepreneurship to occur, strong formal and informal institutions are required (Chowdhury et al., 2018; Dickson & Weaver, 2008; Phelps, 2013; Rodríguez-Pose & Crescenzi, 2008; Urbano et al., 2020). Our research conducted for this paper is part of the rich theoretical and empirical body of new institutional economics. It concerns formal institutions, i.e. bankruptcy and restructuring law, which regulates the treatment of debtors, whether insolvent or threatened with insolvency. The output of the new institutional economics argues that the existing system of incentives or "rewards" or "punishments" in a given society and economy depends to a decisive extent on the quality of formal and informal institutions prevailing at a given time and place (Furubotn & Richter, 2005; Hodgson, 2019; North, 1990; Williamson, 1985).

The development of entrepreneurship and innovation requires special conditions, including formal institutions, i.e. laws that allow for second-chance policies, ensure effective restructuring and, finally, efficient bankruptcy. Legal systems in the area of bankruptcy and restructuring differ despite reforms adopted in the EU member states. Such differences in the legislation of individual member states create legal uncertainty, generate additional costs associated with risk assessment for investors, and lead to poorer development of capital markets and to the persistence of barriers to the effective restructuring of viable EU companies. Differences in bankruptcy law translate to its friendliness or severity towards debtors depending on the context, and thus to the development (or lack thereof) of entrepreneurship and innovation. Moreover, as a result of overly severe bankruptcy law for debtors, entrepreneurs consider bankruptcy a last resort, usually when it is too late (Morawska et al., 2019). This results in a situation where businesses that should have filed a bankruptcy petition a long time ago, so-called zombie companies (Prusak et al., 2019), are still present on the market.

Research on the conditions for the development of entrepreneurship and innovation has a long history. Our research develops the ongoing discussion on these factors; based on this, we have attempted to find an answer to the following research question: is there a relationship between debtor-friendly bankruptcy law and the development of innovation and entrepreneurship? To the best of the authors' knowledge, in previous studies, the analysis of the impact of bankruptcy law on individual variables showing the development of innovation and entrepreneurship was conducted independently. They focused on:

- Identifying criteria for debtor-friendly or creditor-friendly bankruptcy law, as well as hybrid bankruptcy law,
- Searching for relations between measures of entrepreneurship, innovation, corporate performance and the type of bankruptcy regime.

Taking into account the existing academic achievements, it can be assumed that, among other factors, the nature of bankruptcy law has an impact on entrepreneurship development and innovation. It can also be assumed that only some of the described factors influencing the friendliness of bankruptcy law towards debtors have a positive impact on the level of innovation and entrepreneurship. Our aim was to conduct a holistic analysis, taking several factors into account simultaneously. We proposed a descriptive model and put forward the hypothesis according to which: in countries characterised by an effective legal system and at the same time debtor-friendly bankruptcy law, the level of risk acceptance among entrepreneurs is higher, which translates into higher levels of entrepreneurship and innovation. In our opinion, the fact that entrepreneurs are more willing to take risks translates into a higher level of entrepreneurship and at the same time has a positive effect on innovation. The study assumes that in addition to what the law states, how efficiently it is enforced is also important. It is hard to imagine that a bankruptcy law that is friendly to entrepreneurs but ineffectively enforced would have a positive impact on entrepreneurial development and innovation (Fu et al., 2020; Prusak et al., 2018). The following countries were included in the study: Austria, Croatia, Estonia, Finland, Greece, Lithuania, Latvia, Sweden, Germany, Bulgaria, the Czech Republic, France, Spain, Poland, Portugal, Romania, Slovakia, Slovenia, Hungary, Denmark, the Netherlands, Ireland, Italy, the United Kingdom, the United States, Canada, and Australia. The bankruptcy law severity/friendliness index for debtors (BLSI BIS) was developed by the authors and determined based on the analysis of bankruptcy and restructuring laws applicable in each country which was carried out by the co-authors on the basis of legal regulations in force at the end of 2019. As a result, other indicators used in the study were selected in such a way as to fit this period in terms of time.

To verify the research hypothesis, the authors used linear models estimated on the basis of the least-squares method. Additionally, to strengthen the conclusions drawn, the models were assessed in such a way enabling the analysis of causality as defined by Granger based on the two-step process.

Apart from the introduction, the paper also includes the following points: the literature background, research methodology, the research results, and conclusions. The first section briefly presents the research stream within which the studies were conducted. Then, using the method of literature review, the prior scientific output in this area is presented, i.e. the most important research results presenting factors differentiating debtor- and creditor-friendly bankruptcy law and referring to the analysis of relations between the type of bankruptcy law and entrepreneurship as well as innovation level. In the methodology section, a descriptive model is proposed and a research hypothesis is formulated based thereon. The course of research is also described, including: the selection of the research sample, the research period and the applied methods. Subsequently, the results of the research, which are mainly in the form of models, are presented. The article ends with conclusions drawn from the research undertaken, on the basis of which relevant practical and social implications are presented. Moreover, the limitations of the research and proposed directions for further research are also given.

## Literature background

One of the main streams in heterodox schools of economics is the new institutional economics, which studies the impact of various institutions on economic activity. As North (1990) puts it, institutions are certain 'rules of the game in society', which can be both formal and informal. Following North (1990), formal institutions can be seen as:

- a system of property rights,

- laws (enacted, normative),

- regulations (public, social, regarding the real sphere and the financial sector).

Informal institutions, on the other hand, will include:

- culture,

- values (axiological system),

- commonly accepted patterns of behaviour,

- religion and beliefs,—social trust,

- "mental models", i.e. dominant ways of thinking and reasoning in a given society or in particular groups of economic and political actors.

This school is largely interdisciplinary and brings together economics, law, sociology, anthropology, political science and organisation science, among other disciplines. Its main goal is to explain how institutions work, what functions they perform, what changes they undergo and what reforms should be undertaken to achieve a positive economic effect (Klein, 1998; Ménard & Shirley, 2008). Within this stream, one of the main research areas is law & economics, which deals with the study of the impact of legal regulations on economic processes (Cooter & Ulen, 2016). Positive and normative legal theory can be mentioned here. The former shows how current law affects economic processes, while the latter identifies what should be changed in order to improve efficiency and achieve positive effects in the form of economic development. This article attempts to analyse bankruptcy law from the point of view of the effect of its friendliness/severity towards debtors on entrepreneurship and innovation. It has been pointed out that the law does not operate in a vacuum and even good laws require effectively functioning institutions to have a positive impact on the economy. Therefore, this issue fits perfectly into the stream of new institutional economics, with a special focus on the area of law & economics.

In the European Union, measures implementing the second-chance policy have been promoted for several years by reducing the stigmatisation of honest insolvent debtors, simplifying bankruptcy procedures, easing sanctions and enabling them to discharge their debts, among other things. Such activities are supposed to contribute to the development of entrepreneurship, and at the same time increase innovation. However, despite some universal directions of change, the bankruptcy laws of individual EU member states still show many different features. The main criterion differentiating them is the friendliness of bankruptcy regulations towards debtors and creditors. Therefore, in the subject literature, a distinction is made between legal systems that are more debtor- or creditor-friendly and the so-called hybrid systems. Studies aimed at distinguishing bankruptcy systems that are more debtor- or creditor-friendly were carried out, for instance, by Wood (1995a, b), Hussain and Wihlborg (1999), Berglöf et al. (2001), Bliss (2003), Falke (2003), Recasens (2004), Franken (2004), and López-Gutiérrez et al. (2005). In these publications, the authors both presented criteria that differentiate the two systems and attempted to assign specific countries to bankruptcy systems from the point of view of their friendliness to debtors and creditors. However, the classification of countries into individual bankruptcy regimes concerned a relatively small number of countries, apart from the research conducted by Azar (2007) and Morawska et al. (2020). The former proposed the PDI (pro-debtor index) and PCI (pro-creditor index) and assigned 50 countries to more or less debtor- or creditor-friendly systems based on several criteria and data from 2003. The latter (2020) developed the bankruptcy law severity/friendliness index (BLSI) and used it to identify countries with more debtor- or creditor-friendly bankruptcy laws. The study included 23 EU member states, the UK, the USA, Canada, and Australia.

Given the scientific achievement so far, it is possible to distinguish the basic criteria differentiating the two types of systems. In debtor-friendly bankruptcy systems, restructuring is chosen more often than liquidation. These systems are often accused of treating creditors worse than debtors, and managers of an insolvent business unit are more often left in power after bankruptcy than in creditor-friendly systems. This is based on the assumption that managers know the problems of an insolvent company better. Therefore, the Absolute Priority Rule (APR) is often violated in this model. As far as this approach is concerned, social issues, i.e. preserving jobs, play an important role as well. Giving preference to new sources of financing for a bankrupt debtor also constitutes an important criterion differentiating the two systems. The aim of this is to contribute to the maintenance of activity within the business unit. Moreover, it is possible to release debtors from outstanding payments in bankruptcy proceedings, which has a huge impact on the implementation of the second-chance policy. Depending on whether this option is more or less available, such a system is more or less debtor-friendly. Sanctions also play a special role. The more there are and the stricter they are, the less debtor-friendly such law is considered to be.

Contrary to the above concept, the system promoting creditors implies the best possible protection of creditors and the dismissal of current managers from the company's management board, as they are blamed for financial troubles. The liquidation of a company is preferable to its restructuring due to the fact that the latter has negligible effect and, in the case of unsuccessful restructuring processes, companies often return to the path of restructuring, which generates high costs. Other differences concern the regulation of the possibility of making a decision to accept or reject the restructuring plan, creditors voting on the plan, and the so-called automatic stay, which is associated e.g. with the lack of penal interest on liabilities, suspension of court enforcement, etc. as a result of bankruptcy. This system usually ignores reciprocal agreements because they favour one creditor over another. However, on the other hand, it supports the creation of the so-called groups of privileged claims, which, after all, violate the division of claims established before the declaration of bankruptcy. Moreover, it is characterised by severe sanctions for debtors and provides for strict conditions for releasing them from payment of outstanding liabilities within the bankruptcy proceedings, or it does not provide for any such conditions at all.

Other directions of research using the division of bankruptcy systems into those more or less favourable to debtors or creditors consisted of searching for relationships between the measures of entrepreneurship, innovation, enterprise performance and the type of bankruptcy regime. Based on research conducted in four countries (France, Germany, Spain, and the UK), López-Gutiérrez et al. (2005) concluded that in countries with a creditor-friendly bankruptcy system, companies filing for bankruptcy lose more in terms of value than in debtor-oriented countries. According to White (2001, pp. 39–42), the bankruptcy law system has an impact on establishing and running a business in small and medium-sized enterprises. In this case, pro-debtor systems, which are characterised, among other things, by the exemption of insolvent entrepreneurs from debt, are also characterised by a higher level of entrepreneurship – more frequently exempting them from debt results in a higher probability of establishing and running a new sole proprietorship. On the other hand, this factor results in a lower probability of receiving a loan to conduct business activity (banks tighten the criteria due to the lenient treatment of debtors). This relationship is confirmed by research carried out by Cerqueiro and Penas (2017) on a sample of start-ups in the USA. Their analyses show that a higher level of debtor protection provided by U.S. personal bankruptcy law reduces the availability of financing to start-ups and, as a consequence, causes these firms to grow slower and fail more often. According to Landier (2005), the stigmatisation of bankruptcy is one of the main factors determining the development of entrepreneurship. In countries with a higher level of tolerance for bankruptcy and risk acceptance, the development of entrepreneurship is greater. The author distinguishes two models: conservative and experimental ones. The former assumes a high stigma of bankruptcy (stricter bankruptcy law for debtors), whereas the latter assumes greater levels of risk tolerance and acceptance. The experimental model is characteristic of countries focused on innovation and entrepreneurs operating in an aggressive manner, whereas the conservative model is designed for countries where imitation is more prevalent. This model assumes fewer employees will decide to be entrepreneurs, as it is safer for them not to do so. Due to the high costs of bankruptcy in the conservative model, entrepreneurs choose safer projects than in the experimental model. Based on research conducted in 29 countries in the period of 1990–2008, Lee et al. (2011) concluded that there is a positive correlation between the friendliness of bankruptcy law towards entrepreneurs and the level of entrepreneurship measured using the rate of entry of new companies onto the market. In more recent studies, Damaraju et al. (2020) analysed the effects of the interaction between bankruptcy law and culture on the level of entrepreneurial activity. They show that culture can influence the relationship between the severity of bankruptcy law and the level of entrepreneurship. Indeed, due to cultural differences, a positive relationship between the strictness of bankruptcy law and the level of entrepreneurship has been recorded in some countries.

Armour and Cumming (2008) pointed out that in this type of research, apart from the bankruptcy law regulating the insolvency of enterprises, one should take the consumer bankruptcy regulations into account. In the case of small entrepreneurs, e.g. sole proprietorships, it is difficult to separate the company’s assets from the owner's personal property. Due to that fact, in many countries, small entrepreneurs go through bankruptcy proceedings intended for natural persons. Research on the impact of consumer bankruptcy law on entrepreneurship was also conducted by Jia (2015). The results show that entrepreneurs prefer more lenient bankruptcy laws that provide them with greater security. Similar results were also obtained by Fossen (2014). Contrary to previous studies such as that of Estrin et al. (2017), they stated that not all debtor-friendly elements of bankruptcy law have a positive impact on entrepreneurship. Therefore, there is an optimum for debtor and creditor laws which favour entrepreneurial activities.

Innovation is also associated with the development of entrepreneurship. Based on their research, Acharya and Subramanian (2009) concluded that creditor-friendly bankruptcy laws are characterised by an excessive number of liquidations and, consequently, a lower number of innovations compared to more debtor-friendly systems, which support the continuation of business activity. According to Ederer and Manso (2011, p. 94), the pattern of motivation to innovate depends on the level of tolerance for bankruptcy and has an impact on long-term success. Debtor-friendly bankruptcy law has an impact on supporting research and seeking new solutions. If the law is too restrictive for debtors, it may prevent them from conducting such activities, as they will fear the consequences of failure.

**Fig. 1:**
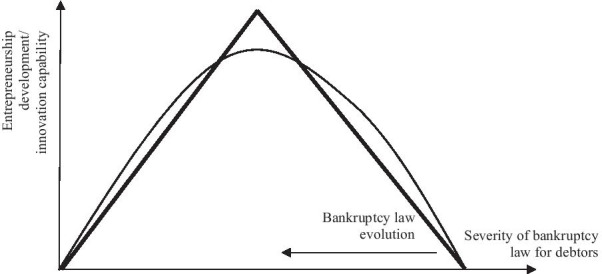
Model of relationships between entrepreneurship/innovation and the level of severity/friendliness of bankruptcy law towards debtors. Source: Authors' own study

Taking the scientific achievements so far into account, it may be assumed that, apart from other factors, the nature of bankruptcy law affects the development of entrepreneurship and innovation. It is also reasonable to assume that only some of the factors described which affect the friendliness of bankruptcy law towards debtors have a positive impact on the level of innovation and entrepreneurship. At this point, it is worth emphasising that, apart from the existing law, how effectively and efficiently it is enforced is also important. It is difficult to imagine that inefficiently enforced entrepreneur-friendly bankruptcy law would have a positive impact on entrepreneurship and innovation (Fu et al., 2020; Prusak et al., 2018). Moreover, the relationship between the friendliness of bankruptcy law towards debtors and the development of entrepreneurship and innovation is unlikely to be linear, as far as the entire relationship is concerned. Assuming that we have a measure of the severity or friendliness of bankruptcy law towards debtors, this relationship would be similar to the upside-down letters U or V, although it would not have to be symmetrical (Fig. 1).

In our opinion, and as suggested by Estrin et al. (2017) among others, the friendliness of bankruptcy law towards debtors is considered to be optimal when the development of entrepreneurship and innovation reaches the highest level. It is hard to imagine that extremely debtor-friendly law would not create negative effects on the market, for example in the form of increased risk of financing of debtors by creditors and the accompanying increase in the cost of raising capital. The directions of changes in the bankruptcy laws, mostly favouring debtors (Frouté, 2007) and promoting the second-chance policy, suggest that the optimum threshold has not yet been exceeded, and one of the countries nearing the threshold is the USA.^1^ Consequently, this may suggest that most countries are now at a stage where the increased friendliness of bankruptcy law towards debtors is accompanied by increased entrepreneurship and innovation.

## Empirical research

### Methodology

**Fig. 2:**
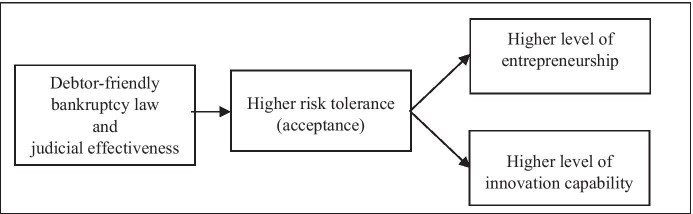
Model of relationships between bankruptcy regimes, risk tolerance, entrepreneurship and innovation. Source: Prusak et al. (2018)

As shown above, bankruptcy law may play an important role and be one of the factors influencing the development of entrepreneurship, innovation, and thus economic growth, among other things. This issue has been observed across the EU, and steps to implement the second-chance policy have been taken; these included e.g. proposing directions for changes in national and EU bankruptcy laws. In previous studies, the analysis of the impact of bankruptcy law on individual variables was conducted independently. Our aim is to conduct a holistic analysis, taking several factors into account simultaneously. Therefore, on the basis of the literature review, a descriptive model was proposed; this is shown in Fig. 2.

According to this model, it was assumed that entrepreneurs are willing to take greater business risks in countries with more debtor-friendly bankruptcy law and a well-functioning legal system. An effective legal system is an essential element because, without it, the law would not be enforced quickly and at relatively low cost. This relationship is confirmed by the research carried out in China (Parry & Long, 2019) and in some European countries (Ippoliti et al., 2015a, b), among others. Ippoliti et al., (2015a, b) have demonstrated that judicial efficiency has an impact on endogenous uncertainty reduction in the markets and consequently has a positive effect on the development of entrepreneurship. Debtor-friendly bankruptcy laws may result in entrepreneurs being less afraid of failure. For example, sanctions imposed for this are relatively small. It is possible to release debt not satisfied in bankruptcy proceedings and to complete such proceedings fairly quickly. Consequently, this has a positive impact on the implementation of the second-chance policy. Entrepreneurs are aware that one failure does not ruin their chances and that they will be able to start their next venture as quickly as possible. Research also shows that entrepreneurs who have experienced failure acquire additional knowledge and experience; as a result, their subsequent businesses are run successfully (Stam et al., 2008). On the other hand, the stigmatisation of insolvent debtors leads to a situation where they rarely decide to restart their businesses (Simmons et al., 2014). The fact that entrepreneurs are more willing to take risks translates into a higher level of entrepreneurship, and at the same time has a positive effect on innovation (Zhao et al., 2021). Entrepreneurs who are not afraid of failure and who, at the same time, have been subject to fewer sanctions are more willing to experiment and seek new solutions. Therefore, it can be assumed that countries with friendly bankruptcy law and a well-functioning legal system will be more oriented towards innovation than imitation.

According to the descriptive model, the following research hypothesis was to be verified in the study:

*In countries characterised by an effective legal system and at the same time debtor-friendly bankruptcy law, the level of risk acceptance among entrepreneurs is higher, which translates into higher levels of entrepreneurship and innovation.*

The research sample mainly comprised the EU member states, the UK, and three non-European countries included in the benchmark. This is due to the fact that the study was supposed to focus on the functioning of bankruptcy systems in the EU member states compared to the selected countries with effective bankruptcy systems. The smallest EU member states and those for which the authors of the publication did not have access to bankruptcy regulations were excluded. Ultimately, the following countries were included in the study: Austria, Croatia, Estonia, Finland, Greece, Lithuania, Latvia, Sweden, Germany, Bulgaria, the Czech Republic, France, Spain, Poland, Portugal, Romania, Slovakia, Slovenia, Hungary, Denmark, the Netherlands, Ireland, Italy, the United Kingdom, the United States, Canada, and Australia.

The analysis was static in nature, i.e. it was conducted on the basis of cross-sectional data available for a similar point in time. This may be regarded as a limitation of the study, but it results from the fact that time-series data were not available for all the variables included in the study. The bankruptcy law severity/friendliness index for debtors (BLSI BIS) was developed by the authors and determined based on an analysis of bankruptcy and restructuring laws applicable in each country which was carried out by the co-authors of the publication. It was conducted on the basis of legal regulations in force at the end of 2019. As a result, other indicators used in the study were selected in such a way as to fit this period in terms of time. The characteristics of the variables describing the areas shown in Fig. 2 are presented in Table 1.

**Table 1:** Description of the variables used in the study

Area of the study	Variable	Description of the variable	Source
Debtor friendliness of bankruptcy law	BLSI BIS (Bankruptcy Law Severity/ Friendliness Index)	This is an indicator developed by the co-authors of the paper. It consists of three components, namely 1) sanctions for failure to file for bankruptcy in the required period or for the debtor's lack of cooperation with the court; 2) regulations regarding the release from debts after the end of bankruptcy or restructuring proceedings; 3) the maximum time allowed for filing for bankruptcy from the moment of the premise Each factor is equally important. It is the arithmetic average of points assigned to each factor based on the analysis of regulations in the field of bankruptcy. Its value is in the range of < 0;1 >, where: values closer to 0 mean that the bankruptcy law of a country is considered to be more debtor-friendly; values closer to 1 mean that the bankruptcy law of a country is considered to be more severe for debtors. More information on this indicator is shown below the table	Morawska et al. (2020)
Judicial effectiveness	Judicial Effectiveness	This indicator is part of the index of Economic Freedom in the field of Rule of Law (https://www.heritage.org/index/pdf/2019/book/chapter2.pdf) It is in the range from 0 to 100. The higher the value, the better a given country is rated	Miller, Kim, & Roberts (2020)
Risk tolerance/ acceptance	1) Risk Acceptance 2) Attitudes towards Entrepreneurial Risk	This indicator is one of the many components of the GEI index. It is between 0 and 1. The higher the index value, the higher the level of risk acceptance among entrepreneurs This indicator is part of the Global Competitiveness Index. It is in the range from 1 to 7. A higher value means a greater appetite for risk among entrepreneurs	Ács et al. (2019) Schwab (Ed., 2019)
Entrepreneurship	GEI (Global Entrepreneurship Index)	It is in the range from 0 to 100. A higher index value indicates a higher level of entrepreneurship in a country	Ács et al. (2019)
Innovation	1) Innovation Capability 2) Resident Applications of Innovations per million people (by origin)	This index is in the range from 0 to 100. The higher the value, the better a country is rated in a given area The higher the value of the variable, the better a given country is rated	Schwab (Ed., 2019) World Intellectual Property Organization (2020)

Source: authors' own study based on the sources shown in the table

The BLSI BIS variable was originally developed by the authors, which is why, apart from other indices shown in Table 1, it requires a more extensive presentation. It was created as a result of the adjustment of the BLSI index originally developed by Morawska et al. (2020). The BLSI index consists of 10 components determining the friendliness/severity of bankruptcy law towards debtors, i.e. 1) sanctions for failure to file for bankruptcy in the required period or for the debtor's lack of cooperation with the court, 2) suspension of bankruptcy proceedings in the event of an application to open restructuring proceedings, 3) number and types of restructuring paths, 4) regulations regarding the release from debts after the end of bankruptcy or restructuring proceedings, 5) accelerated restructuring and/or bankruptcy path for SME entrepreneurs, 6) prepack regulations, 7) debtor in possession regulations, 8) new financing regulations in bankruptcy and restructuring proceedings, 9) the maximum time allowed for filing for bankruptcy from the moment of the premise, and 10) rules for voting among creditors on a restructuring plan or arrangement. Based on a preliminary statistical analysis in the form of assessing the coefficients of the correlation between each of these components and innovation, entrepreneurship, and risk acceptance indices, it was observed that only three of them showed more significant relationships. Therefore, the next step was to propose the BLSI BIS index, consisting of only three factors, namely 1) sanctions for failure to file for bankruptcy in the required period or for the debtor's lack of cooperation with the court; 2) regulations regarding the release from debts after the end of bankruptcy or restructuring proceedings; and 3) the maximum time allowed for filing for bankruptcy from the moment of the premise. The normalised values of these factors determined for each country which was subject to assessment were shown by Morawska et al. (2020). The BLSI BIS index, on the other hand, is the arithmetic average determined on the basis of the normalised values obtained for the three components. From a practical point of view, it is also possible to logically explain why these three factors were selected. An entrepreneur will be less afraid of failure if he/she is able to discharge his/her debts quickly after the completion of bankruptcy proceedings. Moreover, from the entrepreneur's point of view, it is important to know whether he/she is obliged to file for bankruptcy in the case of financial problems and what the deadline for filing the petition is. Failure to meet such a deadline results in the imposition of sanctions. In countries with debtor-friendly bankruptcy laws, it is up to the entrepreneur to decide whether to file for bankruptcy or not, as no penalties are imposed for missing the deadline. It is therefore obvious that in such a case, entrepreneurs will be more willing to experiment. Not all of the factors included in the original BLSI index apply to small entrepreneurs, which constitute the largest population among entrepreneurs overall. Many of these factors are important from the point of view of bankruptcy or restructuring proceedings for medium-sized or larger entities.

The research hypothesis was verified sequentially, i.e. first, the impact of the friendliness of bankruptcy law towards debtors and the effectiveness of legal systems on the level of entrepreneurs’ risk acceptance was assessed. Then, two relationships, i.e. between the variable representing risk acceptance and the variable representing the level of entrepreneurship (path 1) and the variable representing the size of innovation (path 2), were verified. As far as the areas of risk acceptance by entrepreneurs and innovation are concerned, these relationships were assessed using two variables from each area. This approach aims to increase the reliability of the research and, at the same time, the inference. Confirming the expected relationship for more variables representing the same area will show that it will not change regardless of the variable. The analysis was cross-sectional in nature. The study involved different types of models, i.e. linear and non-linear ones. The linear models had better fitting parameters and they were estimated using the least-squares method.

The verification of the hypothesis put forward in the paper was therefore based on two implications, including the accuracy of the antecedent and the consequent. In the case of the first path of the hypothesis, it takes the following form: if in the model for the dependent variable Risk Acceptance, a negative impact of the BLSI BIS value and a positive impact of the variable Judicial Effectiveness are observed (Model 1) and/or in the model for the dependent variable Attitudes towards Entrepreneurial Risk, a negative impact of the BLSI BIS value and a positive impact of the variable Judicial Effectiveness are observed (Model 2), then the high value of Risk Acceptance and/or the high value of Attitudes towards Entrepreneurial Risk are/is reflected in high values of the GEI Index (Models 3 and 4, respectively). In the case of the second path of the hypothesis, the implication to be verified is as follows: if in the model for the dependent variable Risk Acceptance, a negative impact of the BLSI BIS value and a positive impact of the variable Judicial Effectiveness are observed (Model 1) and/or in the model for the dependent variable Attitudes towards Entrepreneurial Risk, a negative impact of the BLSI BIS value and a positive impact of the variable Judicial Effectiveness are observed (Model 2), then the high value of Risk Acceptance and/or the high value of Attitudes towards Entrepreneurial Risk are/is reflected in high values of Innovation Capability and/or Resident Applications of Innovations (Models 5, 6, 7 and 8, respectively). Therefore, a total of eight models were estimated in the study.

The estimation of the models was based on data normalised according to Eq. 1; such a conversion does not change the skewness and kurtosis of the distribution of the converted variables, as well as their mutual correlation (Walesiak, 2014). Moreover, tests of normality of distribution did not provide a basis for rejecting the assumption pertaining to the normal distribution of the analysed variables. Table 2 shows the correlations between the variables used in the study. According to the data presented, the null hypothesis of no correlation was rejected in all cases; however, the significance levels as a result of which the null hypothesis was rejected differed, especially in the case of correlation with the variable BLSI BIS.

**Table 2:** Correlations between the variables used in the study

ITEM	BLSI BIS Index	GEI Index	Risk Acceptance	Attitudes towards Entrepreneurial Risk	Judicial Effectiveness	Innovation Capability	Resident Applications of Innovations
BLSI BIS Index	1	-0.6117008 ***	-0.37133 *	-0.5413382 ***	-0.4469123 **	-0.492663 ***	-0.3361446 *
GEI Index		1	0.76327097 ***	0.78199432 ***	0.87072628 ***	0.88502179 ***	0.70652928 ***
Risk Acceptance			1	0.5173398 ***	0.59229646 ***	0.72770667 ***	0.51450652 ***
Attitudes towards Entrepreneurial Risk				1	0.76594822 ***	0.76236377 ***	0.67431263 ***
Judicial Effectiveness					1	0.81235338 ***	0.67241477 ***
Innovation Capability						1	0.83390961 ***
Resident Applications of Innovations							1

Significance levels *—10%, **—5%, ***—1%

Source: Authors' own study

1 $${z}_{ij}=\frac{{x}_{ij}}{{\mathrm{max}}_{i}\left\{{x}_{ij}\right\}}$$

The models used in the study were estimated using the classical least squares method. It is worth emphasising that the models describe the analysed phenomena for variables taking values from the initial range well. Additionally, to strengthen the conclusions drawn, the models were assessed in such a way as to enable the analysis of causality as defined by Granger based on the two-step process (after Osińska, 2008) – the verification of the significance of individual parameters using Student's t-test (hypothesis H_0_: ${\alpha }_{i}=0$) and combined testing of the causality of vector **X** (independent variables) using the F-test (hypothesis H_0_: ${\alpha }_{1}={\alpha }_{2}=\dots ={\alpha }_{i}=0$).

## Results

According to the hypothesis paths taken, two models (1 and 2) were estimated for the first path to determine the accuracy of the antecedent. The first model comprises Risk Acceptance as the dependent variable and BLSI BIS and Judicial Effectiveness as the independent variables (Table 3).

**Table 3:** Model 1 for the dependent variable Risk Acceptance

ITEM	Coefficients	Standard error	t Stat	p-value
Intercept	0.14327574	0.24885153	0.57574787	0.5701454
BLSI BIS INDEX	-0.122587	0.16729228	-0.7327716	0.47079457
Judicial Effectiveness	0.75206559	0.25667703	2.93000743	0.00732403
R^2^	0.36502153			

Source: Authors' own study

According to the estimates presented, the model is characterised by a relatively poor fit—R^2^ of 36.5%, but given the shape of the hypothesis, the signs before the variables BLSI BIS (negative) and Judicial Effectiveness (positive) are important. Moreover, the form of the model shows that the impact of Judicial Effectiveness on the variable Risk Acceptance is over six times greater than that of BLSI BIS in relation to the absolute value. It is also observed that the variable BLSI BIS is not statistically significant in the model. The second step is to analyse the causality based on the two-step procedure described above. While there is no basis for rejecting the null hypothesis as far as the impact of the variable BLSI BIS is concerned, a significant impact of all variables on Risk Acceptance (F = 6.8982786, p-value = 0.00429643) is observed in the case of the F test. This model will also be used to verify the second hypothesis path.

The second estimated model (Table 4) has a better fit to the data, at a relatively high level of R^2^ = 63.6%. All variables in this model are significant at the significance level of 10%. Similarly to the first model, in this case, the signs before the variables BLSI BIS and Judicial Effectiveness are also consistent with the assumptions of our hypothesis. Furthermore, in theory, changing the normalised value of BLSI BIS by one unit will decrease the value of Attitude towards Entrepreneurial Risk by 0.099 units; in the case of Judicial Effectiveness, the value of the dependent variable will increase by 0.40 units (its impact is nearly four times greater than that of BLSI BIS in relation to the absolute value). With respect to the analysis of causality, the *t* statistic value makes it possible to determine the impact of all the variables used in the model separately at the significance level of 10% (Judicial Effectiveness 1%, BLSI 10%). The total impact of the vector of independent variables on the variable Attitude towards Entrepreneurial Risk (F = 20.982841) may be observed based on the analysis of causality carried out at the second stage using the F-test.

**Table 4:** Model 2 for the dependent variable Attitude towards Entrepreneurial Risk

ITEM	Coefficients	Standard error	t Stat	p-value
Intercept	0.50499287	0.08175004	6.17727951	2.207E-06
BLSI BIS INDEX	-0.099306	0.05495707	-1.8069739	0.0833204
Judicial Effectiveness	0.4011635	0.08432079	4.75758703	7.6917E-05
R^2^	0.63617446			

Source: Authors' own study

**Fig. 3:**
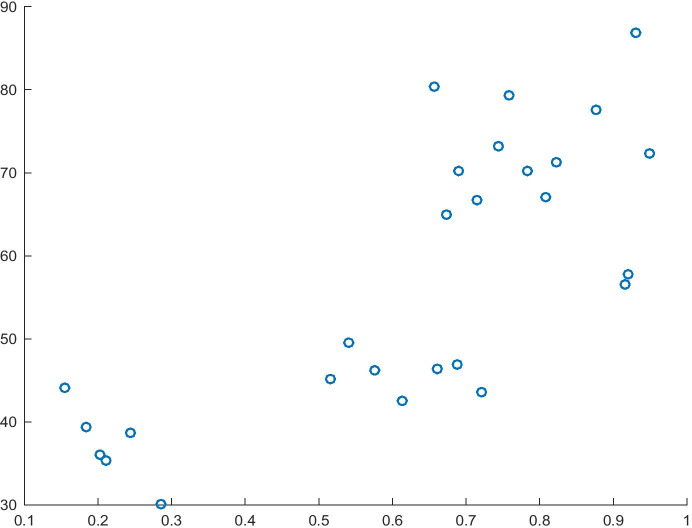
Risk Acceptance (X) and GEI Index (Y). Source: Authors' own study

Model 3 (Table 5) was estimated as the consequent of the implication used to verify the first hypothesis path. In this model, the GEI Index is the dependent variable, whereas Risk Acceptance is the independent variable modelled in Model 1. Based on the model fit of 58% and the significance of the variables used, it can be stated that the model is a good source of knowledge about the GEI index. As estimated, an increase in the normalised value of Risk Acceptance will increase the GEI Index by 0.5755 units. Moreover, the analysis of causality enables us to determine the impact of the variable Risk Acceptance and of the entire model (F statistic = 54.68). The relationship between the dependent variable and the independent variable is shown in Fig. 3.

**Table 5:** Model 3 for the dependent variable GEI Index

ITEM	Coefficients	Standard error	t Stat	p-value
Intercept	0.29711687	0.06535566	4.54615347	0.00012083
Risk Acceptance	0.57550812	0.09742894	5.9069523	3.6491E-06
R^2^	0.58258258			

Source: Authors' own study

**Fig. 4:**
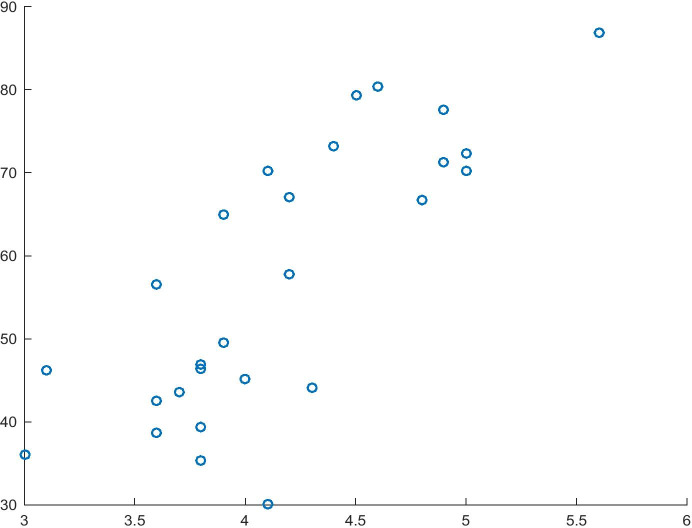
Attitude towards Entrepreneurial Risk (X) and GEI Index (Y). Source: Authors' own study

The model shown in Table 6 is the second component of the consequent of implication developed for the first hypothesis path. The model fit of 61% and the significance of the variables allow the model to be used in the study. As estimated, a change in Attitudes towards Entrepreneurial Risk will increase the normalised value of the GEI Index by 1.36 units. Moreover, the analysis of causality carried out on the basis of the t-test (t = 6.27) and F-test (F = 25.61) makes it possible to reject the null hypothesis that the independent variable and the entire model have no impact on the dependent variable. The relationship between the dependent variable and the independent variable from Model 4 is shown in Fig. 4.

**Table 6:** Model 4 for the dependent variable GEI Index

ITEM	Coefficients	Standard error	t Stat	p-value
Intercept	-0.352007	0.16237359	-2.1678835	0.03989074
Attitudes towards Entrepreneurial Risk	1.35861288	0.21657543	6.27316252	1.4551E-06
R^2^	0.61151511			

Source: Authors' own study

To verify the second path of the hypothesis, Models 1 and 2 were used again (as the antecedent of the implication), whereas estimated models 5–8 were used as the consequent (Tables 7–10).

**Table 7:** Model 5 for the dependent variable Innovation Capability

ITEM	Coefficients	Standard error	t Stat	p-value
Intercept	0.40075366	0.06328785	6.33223742	1.2561E-06
Risk Acceptance	0.50049291	0.09434635	5.30484684	1.697E-05
R^2^	0.52955699			

Source: Authors' own study

**Table 8:** Model 6 for the dependent variable Resident Applications of Innovations

ITEM	Coefficients	Standard error	t Stat	p-value
Intercept	-1.0870256	0.30352213	-3.5813718	0.00143884
Attitudes towards Entrepreneurial Risk	1.84840331	0.40484071	4.56575459	0.00011485
R^2^	0.45469752			

Source: Authors' own study

**Fig. 5:**
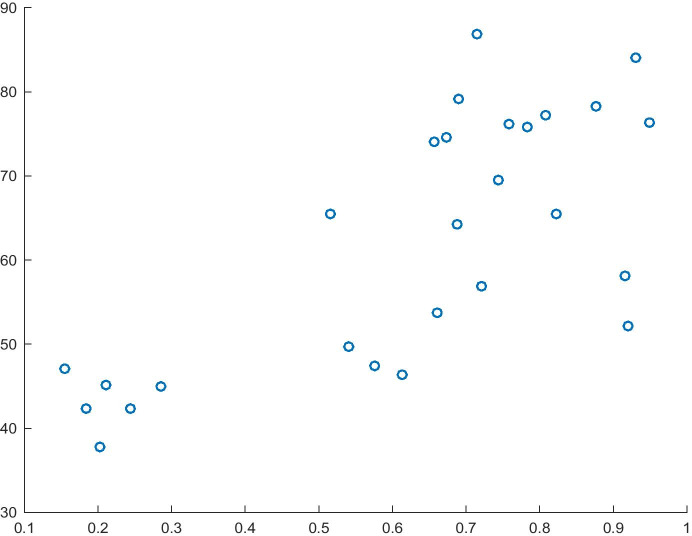
Relationship between Risk Acceptance (X) and Innovation Capability (Y). Source: Authors' own study

As estimated in Table 7, Model 5 shows R^2^ at the level of 53% and significance for all variables. Based on the interpretation of the estimate, it may be stated that an increase of one unit in the normalised value of Risk Acceptance will lead to an increase of 0.50 units in Innovation Capability. Moreover, by analysing the causality, we may also observe the impact of Risk Acceptance as the cause of Innovation Capability and the causality of the entire model (F = 28.1414). This relationship is presented in Fig. 5.

**Fig. 6:**
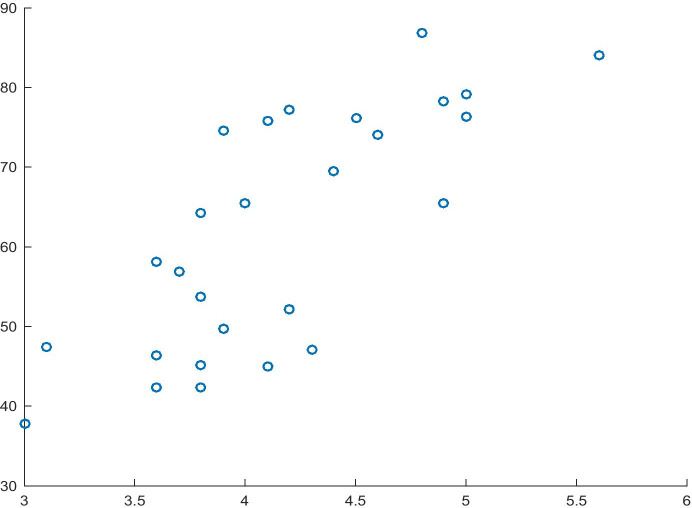
Relationship between Attitude towards Entrepreneurial Risk (X) and Innovation Capability (Y). Source: Authors' own study

The information presented in Table 8 shows that the model has a good fit (R^2^ = 58%), and the independent variable is significant. According to the estimation and the interpretation of the model, an increase in the normalised value of Attitudes towards Entrepreneurial Risk will lead to an increase of 1.21 in the normalised value of Innovation Capability. In addition, the sign before the independent variable coefficient is consistent with the assumption described in the hypothesis. As far as the verification of causality is concerned, the independent variable may be identified as the cause of the dependent variable (t-test), and the causality of the entire model may be verified (F = 34.69). The relationship between the variables, described in Table 8, is shown in Fig. 6.

**Table 9:** Model 7 for the dependent variable Innovation Capability

ITEM	Coefficients	Standard error	t Stat	p-value
Intercept	-0.1835233	0.15378051	-1.1934107	0.24391116
Attitudes towards Entrepreneurial Risk	1.20815665	0.2051139	5.89017438	3.8073E-06
R^2^	0.58119852			

Source: Authors' own study

**Fig. 7:**
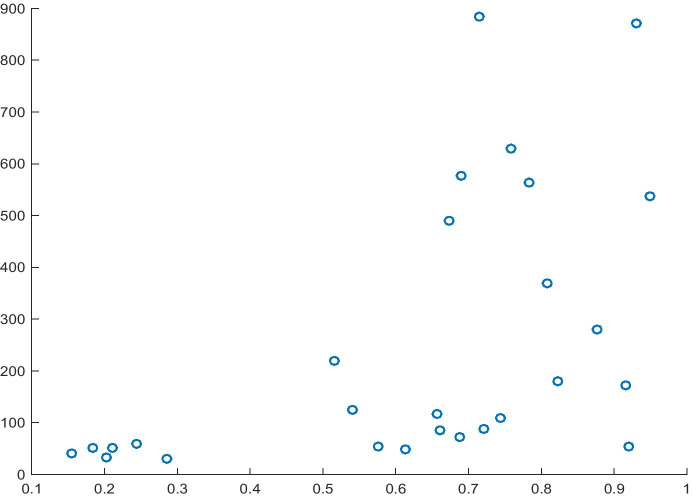
Relationship between Risk Acceptance (X) and Resident Application of Innovations (Y)

In the case of Model 7 (Table 9), the R^2^ value is relatively low (26%); however, its characteristics show that the direction of impact of the variables is always in line with our assumptions (in this case, the positive sign of the Risk Acceptance coefficient), which in turn should enable the verification of the hypothesis. Moreover, based on the analysis of causality, it may be stated that the variable Risk Acceptance and the entire model (F = 9.00) are the cause of the dependent variable. The relationship arising from Table 9 is shown in Fig. 7.

**Table 10:** Model 8 for the dependent variable Resident Applications of Innovations

ITEM	Coefficients	Standard error	t Stat	p-value
Intercept	-0.0972618	0.13685748	-0.7106793	0.48386085
Risk Acceptance	0.6120782	0.20402026	3.00008538	0.00603694
R^2^	0.26471696			

Source: Authors' own study

**Fig. 8:**
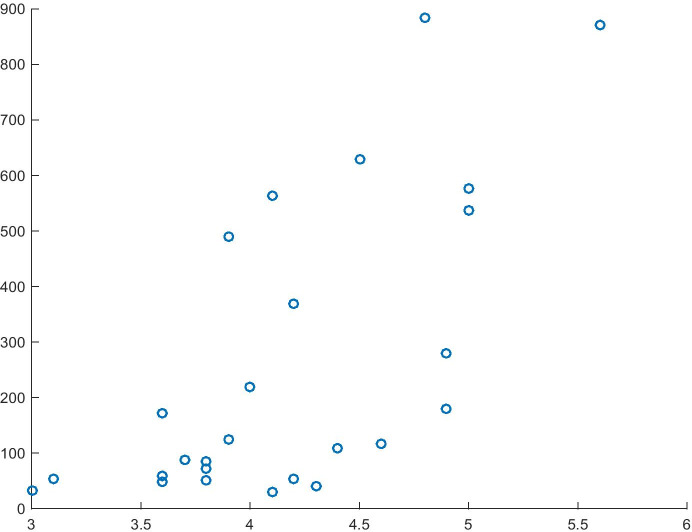
Relationship between Attitude towards Entrepreneurial Risk (X) and Resident Application of Innovations (Y). Source: Authors' own study

The fit of Model 8 (Table 10; R^2^ = 45%) and the significance of the dependent variable allow it to be used for further inference. As estimated, an increase in the independent variable will lead to an increase of 1.85 units in the normalised value of the variable Resident Application of Innovations, which confirms that the direction of impact assumed in the hypothesis is in line with our expectations. Furthermore, the analysis of causality determines both the causality of the independent variable (t-test) and the causality of the entire model (F = 20.85). The analysed relationship is shown in Fig. 8.

## Conclusions and limitations

On the basis of the research conducted, we supported the hypothesis. This means that, in countries characterised by an effective and stable legal system and at the same time debtor-friendly bankruptcy law, the level of risk acceptance among entrepreneurs is higher, which is reflected in higher levels of entrepreneurship and innovation. The following countries were included in the study: Austria, Croatia, Estonia, Finland, Greece, Lithuania, Latvia, Sweden, Germany, Bulgaria, the Czech Republic, France, Spain, Poland, Portugal, Romania, Slovakia, Slovenia, Hungary, Denmark, the Netherlands, Ireland, Italy, the United Kingdom, the United States, Canada, and Australia. The bankruptcy law severity/friendliness index for debtors (BLSI BIS) developed by the authors consists of three components: 1) sanctions for failure to file for bankruptcy in the required period or for the debtor's lack of cooperation with the court, 2) regulations regarding the release from debts after the end of bankruptcy or restructuring proceedings, 3) the maximum time allowed for filing for bankruptcy from the moment of the premise. Thus, as many as seven factors which were not relevant to the analysis were excluded from the basic BLSI measure comprising a total of 10 factors. This means that not all of the components that are characterised by the friendliness of bankruptcy law towards debtors are relevant from the point of view of developing pro-entrepreneurship and pro-innovation policies. This may be due to the fact that the factors included in the original BLSI index and excluded from the BLSI BIS indicator do not significantly affect small entrepreneurs, who constitute the largest share of the overall population of entrepreneurs. Many of these factors are important from the point of view of bankruptcy or restructuring proceedings for medium-sized or larger entities.

The following practical and social implications emerge from the research. Every country should review its national regulations and consider to what extent its bankruptcy/restructuring law favours creditors and to what extent it supports debtors. Apart from the legal provisions themselves, it is also important to apply them effectively in practice. For this purpose, it is worth confronting the so-called *law in books* with *law in action* (Halperin, 2011; Pound, 1910). For example, studies conducted in Poland, Netherlands, Portugal and Italy show, inter alia, that the effectiveness of bankruptcy and restructuring proceedings is negatively affected by: instability of the law, lack of adjustment of the degree of detail in the regulation of proceedings to the size of enterprises, the presence of numerous barriers which lengthen proceedings, no access to modern digital tools, and no specialised administrative staff (Kruczalak-Jankowska et al., 2019). Indeed, a legal system that is effective yet friendly towards debtors with respect to the three components mentioned above may mean that entrepreneurs are less afraid of taking action and thus more willing to take risks and innovate. This, in turn, may be the engine for economic development and progress. Entrepreneurs who calculate their risks through the prism of various sanctions that may be imposed in the legal environment in which they operate may be less efficient and less willing to start a new business in the same or another field of entrepreneurial activities. This study also applies to the implementation of the second-chance policy and shows that honest debtors should not be stigmatised because their experience may contribute to future economic success and the creation of innovations.

The following important limitations to our approach exist as well. First of all, the dataset we use covers only one period—2019—in terms of regulations in force and all other variables used in the models. Moreover, the variables used for model estimation were normalised (formula 1). This is important for understanding the use of the model for an explanation of the dependencies between variables and possible forecasting as it is limited to the data values within the initial sample. The implicit assumption is that, for other countries or years out of our initial sample, the models should work well only for the data that does not exceed the values from the initial sample. Fundamentally it should work for countries that were not initially covered by the research, assuming the dependencies we discovered are universal for the analysed area. This is consistent with the regression assumptions for forecasting where the out-of-sample dependencies can differ from those covered by the estimated models.

In the future, consideration may therefore be given to including panel data; however, one should be aware that this entails a great deal of work and the involvement of many bankruptcy law specialists from different countries. This is due to the fact that it would be necessary to analyse changes occurring in the bankruptcy laws of the analysed countries over a longer period of time. It should be borne in mind that only in the case of a few of the analysed countries were translations of bankruptcy acts into a foreign language, e.g. English, available, which made the research much more difficult. In addition, it would be worthwhile to extend research to new countries, including those with a lower level of economic development. Extending the model proposed in the paper to include cultural variables could also be considered. In addition to the analyses conducted at the macro level, qualitative research in individual countries could be considered to illustrate the perception of insolvency law by entrepreneurs themselves and its impact on risk acceptance, entrepreneurship level and innovation.

## Data Availability

Morawska, S., Prusak, B., Banasik, P. Pustułka, K., & Groele, B. (2020). Bankruptcy Law Severity for Debtors: Comparative Analysis Among Selected Countries. *European Research Studies Journal,* XXIII (Special Issue 2), 659–686. https://doi.org/10.35808/ersj/1847. Miller, T., Kim, A.B., & Roberts J.M. (2020). *2020 Index of Economic Freedom.* The Heritage Foundation, https://www.heritage.org/index/pdf/2020/book/index_2020.pdf. Ács, Z.L., Szerb, L., Lafuente, E., & Márkus, G. (2019). *Global Entrepreneurship Index 2019*. The Global Entrepreneurship and Development Institute. Schwab, K. (Ed.) (2019). *The Global Competitiveness Report 2019.* World Economic Forum, http://www3.weforum.org/docs/WEF_TheGlobalCompetitivenessReport2019.pdf. World Intellectual Property Organization. Last updated April 2020. WIPO IP Statistics Data Center, https://www3.wipo.int/ipstats/index.htm.

## Footnotes

1 U.S. bankruptcy law is considered one of the most effective in the world (Jackson & Skeel, 2013, pp. 5–6). Moreover, it is also defined as debtor-friendly (Brouwer, 2006, pp. 11–12).

## Declarations

### Code availability (software application or custom code)

Excel, OxMetrics, Matlab.
